# Treatment of Puerperal Infection

**Published:** 1904-07-30

**Authors:** 


### TREATMENT OF PUERPERAL INFECTION.

Cabanes [1] records the results of treating puerperal infection with iodised gauze. He prepares the dressing by steeping gauze in a solution of sterile water and potassium iodide in which 4 per cent. of iodine has been dissolved. The uterus is first douched with an antiseptic solution, and, if necessary, curetted with a blunt instrument. The uterus is then loosely plugged with the prepared gauze, and the vagina is douched out and iodoform gauze is placed in contact with the cervix to absorb leakage of iodine and prevent its injuring the vagina. This dressing is changed every 12 hours.

[1] Medical Press, July 13, 1904.

